# Insect stereopsis demonstrated using a 3D insect cinema

**DOI:** 10.1038/srep18718

**Published:** 2016-01-07

**Authors:** Vivek Nityananda, Ghaith Tarawneh, Ronny Rosner, Judith Nicolas, Stuart Crichton, Jenny Read

**Affiliations:** 1 Institute of Neuroscience, Henry Wellcome Building for Neuroecology, Newcastle University, Framlington Place, Newcastle Upon Tyne, NE2 4HH, United Kingdom; 2 M2 Comportement Animal et Humain, École doctorale de Rennes, Vie Agro Santé, University of Rennes 1, 35000, Rennes, France

## Abstract

Stereopsis - 3D vision – has become widely used as a model of perception. However, all our knowledge of possible underlying mechanisms comes almost exclusively from vertebrates. While stereopsis has been demonstrated for one invertebrate, the praying mantis, a lack of techniques to probe invertebrate stereopsis has prevented any further progress for three decades. We therefore developed a stereoscopic display system for insects, using miniature 3D glasses to present separate images to each eye, and tested our ability to deliver stereoscopic illusions to praying mantises. We find that while filtering by circular polarization failed due to excessive crosstalk, “anaglyph” filtering by spectral content clearly succeeded in giving the mantis the illusion of 3D depth. We thus definitively demonstrate stereopsis in mantises and also demonstrate that the anaglyph technique can be effectively used to deliver virtual 3D stimuli to insects. This method opens up broad avenues of research into the parallel evolution of stereoscopic computations and possible new algorithms for depth perception.

Stereopsis – the ability to compare the images seen by each eye and use the disparity between them to perceive depth – is a marvellous feat of visual computation^1^. It was recently described as “one of the most productive model systems for elucidating the neural basis of perception”^2^. A large body of work has investigated and shed light on the mechanisms by which humans and other primates achieve stereopsis^1^,^3^,^4^,^5^. It has also been demonstrated in other vertebrate species including horses^6^, falcons^7^, owls^8^ and toads^9^. The overwhelming majority of work investigating mechanisms of stereopsis has, however, thus far been restricted to primates, cats and owls. Stereopsis has been demonstrated in only one invertebrate – the praying mantis^10^,^11^. It has been suggested that the computational mechanisms underlying stereopsis in insects must be fundamentally different from those in vertebrates^12^, but at the moment this remains speculation due to technical limitations. A major barrier to investigating stereo mechanisms in insects has been the lack of stereoscopic displays like those used with humans and other vertebrates^6^,^7^,^8^,^9^,^13^,^14^, which enable independent images to be presented to left and right eyes. Circumstantial evidence of mantis stereopsis was first provided by the observation that many of their behaviours require binocular vision^15^,^16^. The experiments that first clearly indicated insect stereopsis made use of two prisms placed before the mantises’ eyes, through which they viewed a blowfly that moved towards them on a mechanical apparatus^10^. By using prisms with different powers, this experiment demonstrated that mantises could be fooled into making systematic errors in depth judgment that corresponded to the specific binocular disparity cues provided by the prisms. A final indication of stereopsis – the only one using virtual stimuli – was made by looking at how mantises strike when presented with two competing targets on a computer screen. When physical occluders blocked each eye’s central view of one of the targets, the peripheral crossed monocular cues provided an illusion of depth through disparity^11^. None of these methods, however, allowed for manipulation of binocular disparity cues independently of other properties of the visual stimulus - such as size - or for more complex stimuli such as the random-dot patterns that have revolutionized the study of primate stereopsis^17^,^18^,^19^.

As a result, it has been impossible to explore the mechanisms underlying insect stereopsis. Given the independent evolution of vision in insects^20^ and the evolutionary distance between mantises and all the other animals in which we see stereopsis, studying the mechanisms of stereopsis in insects is important in two ways. The first is that it enables us to explore whether nervous systems in vastly divergent evolutionary lineages have evolved convergent or divergent solutions to the same complex problems. The second interesting possibility is that insects might have evolved novel mechanisms for stereopsis that might be easier to adapt into robotic or computational systems of depth-perception, given the relative simplicity of their nervous systems. In this study we therefore attempted to develop a new technique that would enable the use of arbitrary binocular stimuli to investigate insect stereopsis.

## Results

**Figure 1:**
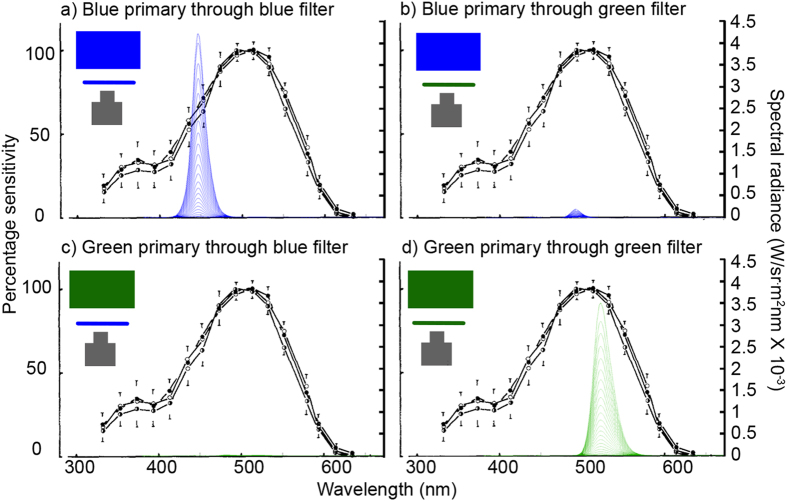
Filter design for the anaglyph 3D glasses. Spectral radiance of light across different wavelengths as transmitted through the filters (y-axis on the right) compared to the mantis spectral sensitivity curve reproduced from Rossel (1979)^27^ (y-axis on the left). Different individual curves show spectral radiance measured at increasing digital driving levels. (**a**) Light from the blue primary transmitted through the blue filter. (**b**) Light from the blue primary transmitted through the green filter. (**c**) Light from the green primary transmitted through the blue filter. (**d**) Light from the green primary transmitted through the green filter.

The most widely-used contemporary 3D technology for humans uses circular polarization to separate the two eyes’ images. We tested this approach for the praying mantis, but found that it failed, apparently due to high crosstalk for stimuli which are not directly in front of the mantis^21^ (see Supplementary Material). We therefore turned to “anaglyph” 3D, as in old-style red/blue 3D glasses, where left and right images are separated by spectral content. Since red light is poorly visible to mantises, we used green/blue glasses (Fig. 1). We selected an LED monitor with unusually narrow-band spectral output in the green and blue wavelength ranges and used colored filters which transmitted light in the same two ranges (Fig. 1, see Methods for details).

### Assessing interocular crosstalk

A problem affecting all filter-based stereoscopic display systems is “crosstalk” between the two eyes, i.e. when stimuli meant for one eye are also visible in the other eye^22^. These “ghost” images do not have the intended disparity and degrade the depth percept, although in humans, stereoscopic depth illusions can survive relatively high levels of crosstalk^23^,^24^,^25^,^26^. We assessed the degree of crosstalk in our stereoscopic display in three ways: with physical measurements of the transmitted light, through electroretinograms and behaviorally (see Methods for details and for definition of crosstalk.)

**Figure 2:**
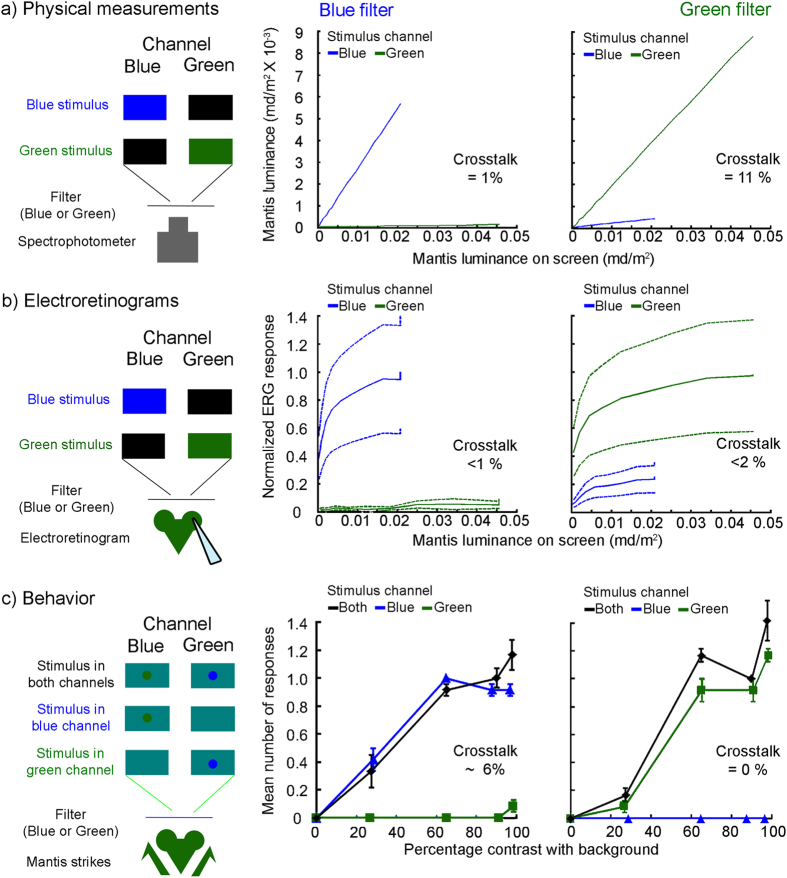
Spectral content crosstalk measurements. Columns correspond to the different filter types –blue or green. Lines represent measurements when light was output in either the blue or green channels. (**a**) Spectrophotometer measurements. Effective luminance at a given digital driving level is obtained by weighting the spectral radiance curve, Fig. 1, by the mantis spectral sensitivity at each wavelength^27^, and then integrating to find the total area under the weighted curve. The analogous calculation using human spectral sensitivity gives luminance in units of candela/m^2^; we define mandela/m^2^ as the unit for mantis luminance. For each primary, luminance measured through the specified filter is plotted against the luminance measured with no filter. (**b**) Electroretinograms in response to blue and green light flashes of different intensities viewed through the blue and green filters. The electroretinogram data represent the mean normalised response with standard error. (**c**) Mean (± S.E.) number of responses (strikes + tensions) to targets presented in the blue (blue line and triangles) or the green (green line and squares) channel or both (black line and diamonds). Note that when the (dark) stimulus is presented in the blue channel, the stimulus appears green on the screen and vice versa. Through the appropriate filters, however, all stimuli appeared dark against a light background. See text and methods for further details.

For the physical crosstalk, we used a spectrophotometer to measure the light transmitted through both blue and green filters when the monitor displayed green and blue stimuli, and weighted this by the mantis spectral sensitivity^27^. We found a relatively low level of physical crosstalk for both the green and the blue filters (<1–11% at maximum luminance; Fig. 2a) after weighting by the mantis spectral sensitivity. To measure the crosstalk using electroretinograms we allowed light from the monitor to reach the eye of the mantis through either a blue or a green filter while presenting flashing light stimuli of different brightness in either the green or the blue spectral range. The physiological crosstalk measured in this way was also very low (<1–2% at maximum luminance; Fig. 2b): the response even to maximum-luminance stimuli presented in the “wrong” channel was less than the response to minimum-luminance stimuli presented in the correct channel.

To assess the spectral crosstalk behaviorally, we placed the mantis 3 cm from the LED monitor, with either a green or a blue filter in front of it, covering the centre of the screen where the stimulus came to rest. The monitor’s blue and green pixels were set to a high value across the whole screen. The stimuli were dark disks spiralling in from the edge to the centre of the screen. These resemble prey to the mantis, so that when presented at this close range they reliably elicit strikes in normal, 2D viewing. We presented these stimuli in random order in either the blue or green channel, or both (see Fig. 1 and Methods for details). To present a stimulus in the blue channel, we dimmed the blue pixels in the target region, so the screen displayed a green target on a cyan background. Viewing this through the blue filter, the mantis would see a dark target moving across a bright background, and would strike at it. If the blue filter succeeded in blocking all light from green pixels, a target presented in the green channel (i.e. a blue target on a cyan background) would be invisible when viewed through the blue filter. Thus, in the ideal case of zero crosstalk, the mantis would never strike at a target presented in the wrong channel. Conversely, stimuli presented either in the same channel as the filter, or in both channels at once would appear identical, and thus elicit the same rate of strikes. As Fig. 2c shows, our results are close to this ideal case, with crosstalk <6%. There were significantly different responses to the three different channel conditions (Green Filters: Repeated measures ANOVA, F(2,6) = 69.16, Partial η^2^ = 0.958, P < 0.001; Blue Filters: Repeated measures ANOVA, F(2,6) = 39.414, Partial η^2^ = 0.929, P < 0.001). The mean response to same-channel stimuli was significantly different from the response to wrong-channel stimuli, with the latter being close to zero (Fig. 2c; Green Filters: Repeated measures planned contrasts: Mean difference = 0.617, P = 0.002; Blue Filters: Repeated measures planned contrasts: Mean difference = 0.633, P = 0.001). The mean response to same-channel stimuli was not significantly different from the mean response to both-channel stimuli (Fig. 2c; Green Filters: Repeated measures planned contrasts: Mean difference = 0.133, P = 0.161; Blue Filters: Repeated measures planned contrasts: Mean difference = 0.033, P = 0.761). As Fig. 2c shows, mantises will strike at even relatively low-contrast stimuli presented in the correct channel (mean rate of 0.25 strikes per trial for stimuli of ~28% contrast), while they virtually never strike even at high-contrast stimuli in the wrong channel (mean of 0.045 strikes/trial for stimuli of 100% contrast). This suggests that any “ghost” images due to crosstalk must be nearly invisible to the mantis.

### Demonstrating illusory 3D depth

**Figure 3:**
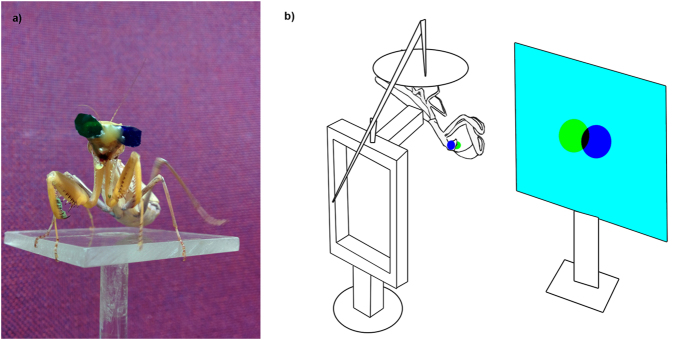
Experimental set-up. (**a**) Mantis fitted with the experimental 3D colored glasses. (**b**) The “insect 3D cinema” for the display of stereoscopic stimuli to the mantis.

Given these encouraging results regarding effective crosstalk, we examined whether the mantis would experience illusory stereoscopic depth due to binocular disparity cues presented via spectral filters. We crafted miniature 3D glasses by cutting out pieces of filter, about 7 mm in diameter. We fixed these onto the mantis’ face with a mixture of beeswax and rosin, a different color filter in front of each compound eye (Fig. 3a). We could now manipulate the binocular disparity cues presented by a virtual, computer-generated stimulus. Again, we used a dark prey-like target moving on a bright background. This time, we presented the target in both channels. The mantis’ 3D glasses ensured that (apart from cross-talk) each compound eye could see only the target presented in that eye’s channel. In this experiment, the mantises were placed further back, so that the monitor was outside their catch range.

**Figure 4:**
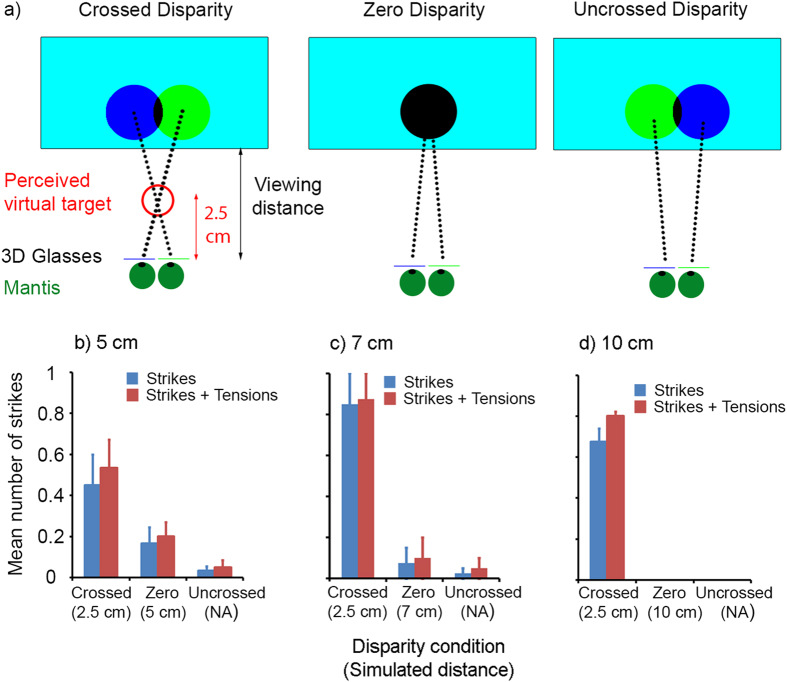
Illusory depth experiment. (**a**) Stimulus geometry for each of the three disparity conditions. (**b–d**) Mean proportion of responses to swirling stimuli across different disparity conditions and viewing distances (indicated above each plot). Blue bars indicate strikes; red bars indicate the sum of strike and tensions. Error bars indicate standard error. Different disparity conditions were interleaved within an experiment; different viewing distances were blocked.

In the “crossed-disparity” condition (Fig. 4a, first panel), the target locations were offset horizontally, contralateral to the eye in which it was visible. In species with stereopsis, like humans, this binocular geometry causes an illusory percept of a target floating in front of the screen, in the position where a real object would create the same retinal images. For each physical distance of the monitor, we computed the crossed disparity so as to simulate a target 2.5 cm in front of the mantis (see Table 1). In the “zero-disparity” condition (Fig. 4a, second panel), the target location was the same in the two channels. This is the same as a normal, 2D display, where both eyes necessarily see a visual stimulus at the same position on the screen. The “uncrossed-disparity” condition (Fig. 4a, third panel) had the same target disparity as in the crossed-disparity condition but with left and right-eye images exchanged. This acted as a control to check that the mantis responses to the crossed-disparity condition reflect the binocular geometry, not merely monocular target locations. We interleaved the three disparity conditions randomly from trial to trial, at physical viewing distances of 5, 7 and 10 cm.

For every physical viewing distance, the mantises struck far more often in the crossed disparity condition compared to the zero disparity and uncrossed disparity condition (Fig. 4b–d: Repeated measures ANOVA, main effect of disparity, F(2,6) = 41.579, Partial η^2^ = 0.933, P < 0.001; Planned contrasts: Mean differences = 0.617, 0.692, Ps < 0.01). In fact, the physical viewing distance had no effect (Repeated measures ANOVA, main effect of distance F(2,6) = 1.015, Partial η^2^ = 0.253, P = 0.417; interaction disparity*distance, F(2,6) = 1.337, Partial η^2^ = 0.308, P = 0.312) . This indicates that mantises were sensitive to the simulated distance of the virtual object, which was the same in all crossed conditions, and not to the physical distance. The effect of disparity was most dramatic when the physical viewing distance was 7 or 10 cm, well beyond the mantises’ catch range. Here, the mantises almost never struck at the zero and uncrossed disparity trials, while they struck on over half of trials in the crossed disparity condition (Fig. 4b–d). This is convincing evidence that the mantises perceived the target at the simulated distance indicated by the disparity and is the first definitive proof that mantises have stereoscopic vision using virtual 3D stimuli.

## Discussion

An increasingly sophisticated combination of physiology, behavioral and computational techniques are being brought to bear on stereoscopic vision as a model perceptual system^2^. In other areas, insights from simpler invertebrate systems have been hugely valuable in understanding primate vision. Hartline’s discovery of lateral inhibition in the horseshoe crab^28^ and Reichardt’s model of motion detectors in the weevil^29^ are just two examples. No such transfer has been possible in stereopsis, since no one has been able to present arbitrary stereoscopic stimuli to invertebrates. We here show that blue/green anaglyph 3D glasses successfully achieve this for the praying mantis. This establishes the first insect model suitable for a systematic exploration of stereopsis.

Our results were by no means self-evident prior to our experiments. Indeed, similar experiments using polarized filters failed (see Supplementary Material). Stereo vision in insects might be processed in dramatically different ways to human stereo vision and we could not automatically expect our approach with this method to yield results. Anaglyph 3D technology designed for humans often suffers from high levels of interocular crosstalk^30^. By choosing an appropriate monitor and filters, we have been able to achieve low levels of crosstalk in our experiments (Fig. 2). A further serious disadvantage of anaglyph 3D for humans is that the color mismatch creates rivalry and reduces depth sensitivity^31^. Unusually among insects, the available evidence suggests that mantises are monochromatic^27^,^32^ (Fig. 1). Even in polychromatic insects, color information is not used in motion processing^33^ and stereopsis might similarly be processed Independently of color given that mantises use it to target moving prey. Thus, it seems likely that mantis stereopsis is blind to spectral differences between the two eyes’ images. However, colored filters also introduce differences in luminance. In our experiments, we used the same digital driving levels for blue and green channels. Given our monitor and the spectral sensitivity determined for other mantis species^27^,^34^, this would translate into unequal perceived luminance (Fig. 2a). Our data suggest that we should have reduced the luminance in the green channel by a factor of roughly 0.7 to equalize luminance across both channels. We nonetheless observed clear and unequivocal responses demonstrating stereoscopic perception. It therefore appears that insect stereopsis, like human, is fairly robust to substantial interocular differences in luminance and contrast^24^,^25^,^26^. For example, for high-contrast stimuli containing high spatial frequencies, like our disk, human stereoacuity is virtually unaffected by a 10-fold interocular contrast difference^23^,^26^ We do not know if mantis stereopsis shows the same robustness or how this depends on spatial frequency, but this would be worth investigating.

We have used our insect 3D cinema to provide clear and dramatic proof of stereopsis in insects. This technique opens up broad new avenues of research. It is now possible for the first time to show insects arbitrary binocular stimuli, such as the random-dot stereograms containing targets that are invisible without stereopsis. By investigating what image features are required for insect stereopsis to function, we can start to tease apart the algorithms involved. Understanding stereopsis in a system as simple as an insect has the potential to provide new insights about human vision, reveal the convergent evolution of neural algorithms and inspire the development of simple, robust stereo algorithms for robotics.

## Methods

### Experimental subjects

All experiments were carried out on adult female mantises of the species *Sphodromantis lineola*. The mantises were housed in individual plastic boxes (7 cm length × 7 cm breadth × 9 cm height) which allowed for ventilation via holes in the lids. Mantises were free to move within the boxes. The boxes were stored in an insect housing facility maintained at 25 °C. The boxes were cleaned and misted with water twice a week and each individual was fed with a live adult cricket twice a week.

### Stimuli and display

Stimuli were displayed on a DELL U2413 LED monitor, chosen because it has narrowband spectral output in the blue and green regions of the spectrum. The screen is 51.8 cm wide by 32.4 cm high, with a resolution of 1920 × 1200 pixels and a 60 Hz refresh rate. All stimuli were custom written in Matlab (Mathworks) using the Psychophysics Toolbox^35^,^36^,^37^. Our standard stimulus for behavioral experiments was the “spiraling disc” target presented on a bright background. This was a dark disc which appeared peripherally and spiraled in towards the center of the screen over the course of 5 s. On reaching the center, it stayed there quivering with a subtle jerky motion for a further 2 s, before vanishing. We have found that this stimulus reliably elicits strikes when presented within the mantis’ catch range at a size of 1 or 2 cm^38^.

### Preparation and fixing of 3D glasses

We used green and blue filters distributed with a preprint of a previously published paper^39^. The 3D glasses were two teardrop shapes, one cut out from each filter, with a maximum diameter of about 7 mm (Fig. 4a). The filters had transmission peaks at 432 and 528 nm which are in the range of mantis spectral sensitivity^27^,^34^ (Fig. 1). When used with the DELL U2413 monitor, the light transmitted through each channel had almost no spectral overlap, except for a small leakage from the blue primary into the green range (Figs 1c and 2a). We can also estimate that, at the maximum digital driving level, the effective luminance of the blue primary viewed through the blue filter was about 70% that of the green primary viewed through the green filter (Fig. 2a).

In both techniques, prior to fixing the glasses, the mantis was first placed in its cage in a freezer (Argos Value Range DD1-05 Tabletop Freezer) for 5–7 minutes in order to immobilize it. Subsequently, the mantis was held in place under a microscope lens by holding down its legs with Plasticine® modelling clay (Flair Leisure Products plc). Previously cut out glasses were then affixed on the mantis with a mixture of beeswax and rosin that was melted and applied using a Denta Star S ST 08 wax melter. In addition to the glasses, a small component, designed for electronics, was glued onto the base of the mantis’s pronotum. This could later fit into a counterpart on the experimental stand and hold the mantis in place during experiments without restricting the movement of its head and forelimbs. After the glasses and the component were fixed, mantises were released and placed back in their cages where they were allowed a minimum of 24 hours to recover. Experiments were only carried out after this rest period.

### Experimental set-up

For experiments, the mantises were attached to the frame of the experimental stand^10^,^11^ (Fig. 3b) with the components described above. The stand consisted of a Perspex® frame that could be easily rotated and raised vertically and was fitted with a card disc for the mantis to rest on. The disc was held in place by a copper rod with a weight on the opposite end and gave the mantis a feeling of flexibility and mobility^11^. The mantises were placed upside down, their preferred position, and could then hold onto the disc with their legs (Fig. 3b). Once the mantis had been placed on the stand, the stand was placed in front of the monitor at the appropriate viewing distance.

### Behavioral data recording and analysis

All behavioral responses were recorded using a Kinobo USB B3 HD Webcam (Point Set Digital Ltd, Edinburgh, Scotland) placed directly beneath the mantis. The output of the camera was fed to a RM Expert 3040 computer where the video files were saved. Video recording was synchronized with the stimulus presentation and started when each stimulus first appeared on the screen, ending when the stimulus disappeared. The camera was placed at a position that ensured that the monitor was outside the visible range of the camera. The recordings could thus be analyzed blind to the stimulus. We coded the recordings for two different responses of the mantises: strikes and tensions. strikes involved a rapid release of the forearms typically in an attempt to capture perceived prey^38^ and tensions involved a tensing of the forearms in preparation for a strike that was eventually unreleased. We used the sum of strikes and tensions as our behavioral measure in the crosstalk experiments with around 87% of responses being strikes. The results did not change significantly if we used only strikes as our measure. In one experiment using polarizing filters (see Supplementary Material) we also used a third behavioral response: tracks. These were sharp, saccadic head movements that follow sudden movements in the visual field of the mantis^38^, The computer independently recorded the order of presentation of the stimuli, so that after manual coding we could assign the response to the correct condition.

### Assessing interocular crosstalk

In a stereoscopic system, physical crosstalk is defined as the amount of luminance leaking through the “wrong” channel (e.g. green primary viewed through blue filter) relative to the luminance passing through the correct channel (e.g. blue primary viewed through blue filter):


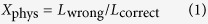


In Fig. 2a, we evaluate this at the maximum luminance level. Recall that the definition of luminance takes into account the spectrum of the light emerging from the filter and the spectral sensitivity of the organism.

The definition of physiological and behavioral crosstalk additionally takes into account non-linearities in the organism’s response to luminance. For example, if at the maximum luminance of our display, the leak luminance is below threshold for eliciting a retinal response, then physiological crosstalk is effectively zero. To measure this, we compare the response to the maximum luminance presented in the wrong channel, and ask how much luminance must be presented in the correct channel to elicit the same response:


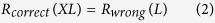


where *X* is the crosstalk ratio and *L* is the maximum luminance. Where the response is a linear function of luminance, this is the same as the previous expression. Thus to compute the crosstalk in Fig. 2b,c, we draw a horizontal line from the value of the “wrong channel” response at 100% input, and see where this intersects the “correct channel” curve.

#### Physical measurements (spectrophotometer)

To measure the physical crosstalk of the filters, we fixed a filter on the monitor directly in front of the Konica Minolta CS-2000 spectrophotometer such that it covered the entirety of the measurement area of the spectrophotometer. Measurements were subsequently made in complete darkness. A custom-written program then displayed a square target on the monitor that covered the entire measurement area of the spectrophotometer. The background of the display was kept white (i.e. the digital driving level was set to the maximum value, 255 in our 8-bit system, for all three primaries). The target square was presented in the blue or green primary only. The digital driving level in that primary was increased from 0 to 250 in 25 steps, with a spectrophotometer measurement taken at each step, while the digital driving levels in the other two primaries were kept at zero. These measurements were repeated for a blue/green target viewed through the blue/green filter.

#### Physiological measurements (electroretinograms)

The mantises were immobilized and tethered on a custom made holder. The mantises viewed the monitor through a cylindrical tunnel built from black cardboard (radius 6 cm, length 8.5 cm) that was blocked with card on either side except for a rectangular window (1.5 × 3 cm) for the mantis and a square window (of side 3 cm) that fit onto the monitor. A filter, either blue or green, was placed between the mantis and the tunnel. The construction prevented the photoreceptors being excited by reflections in the experimental setup. We used wick-electrodes or borosilicate micropipettes drawn on a microelectrode puller (Sutter Instruments P-97, USA) for the recordings. Responses were amplified (BA-03X amplifier; npi electronic, Tamm, Germany) digitized (CED1401 micro; Cambridge Electronic Design), and stored using a PC with Spike2 software (Cambridge Electronic Design, Cambridge, UK). We recorded electroretinograms in response to light flashes of increasing amplitude presented in either the blue or green primary of the monitor. The stimuli were slightly bigger than 3 × 3 cm window visible to the mantis, so they covered it completely. The results of Fig. 2a were used to calculate the luminance corresponding to each digital driving level. One stimulus sequence comprised 10 light flashes, each lasting 500 ms and separated by five or eight seconds of darkness. A new stimulus sequence was started manually about every 10 seconds. After several repetitions, the filter was changed and the procedure was repeated. In some electroretinograms, the filters were changed again and the procedure repeated. Amplitudes in response to light flashes were defined as the average potential over the last 100 ms of a 500 ms light flash, minus the average potential over the 100 ms before the flash onset. Electroretinograms were obtained from two recordings from each of two insects. The response was normalized for each recording and then averaged for each insect. The results in Fig. 2b show this mean response averaged across recordings and insects normalized such that the maximum response in each channel is 1. The signal response at the minimum value tested is greater than the maximum leakage response, so we cannot compute crosstalk exactly but only state that it is <2%.

#### Behavioural measurements

Mantises, not fitted with glasses, viewed stimuli through a blue or green colored filter of dimensions 4.5 by 4.5 cm. The filter was fixed directly to the screen, at the centre of the screen over the location where the stimulus comes to rest. The mantises were placed 3 cm from the monitor, a distance at which they will reliably strike at computer-generated stimuli presented on a 2D screen in the normal way. “Spiraling disc” stimuli, with a diameter of 1 cm on the screen, were then presented in either only the green or blue color channels or both. For the background, the digital driving level was set to 128 in both the blue and green primaries. To present a dark target in the blue or green channel, the digital driving level of that primary was reduced to one of five possible values (0, 32, 64, 96, 128) which were randomly interleaved across trials. The last value corresponds to no stimulus, since the “target” was the same as the background. Again, Fig. 2a was used to translate each digital driving level into the luminance of the background, B, and target, T. Figure 2c shows response rate as a function of target contrast relative to the background, (B−T)/(B + T). In one experimental run each combination of the three channel conditions (blue, green, both) and five contrast conditions was presented three times in random order for a total of 45 trials per experimental run. Four mantises were tested with each of the two filters and with 3 trials each for every combination of contrast and disparity condition. Note that in this experiment, both eyes always viewed the same stimulus, so there was no binocular disparity. The target was always presented at the same location in blue and green channels.

### Demonstrating illusory 3D depth

In this experiment, the mantises were fitted with 3D glasses so that different images could be presented to the two eyes; there was no other filter in front of the monitor. Each eye was fitted with one or the other color filter and across animals we alternated whether the left or the right eye was fitted with the blue or green filter. The stimulus was again the “spiraling disc” target, this time presented with maximum contrast in the three disparity conditions as described above. As before, the digital driving level in the background was set to 128 in both the blue and green primaries. In the zero disparity condition, the disc was black in both channels (RGB = [0 0 0]). In the crossed and uncrossed disparity conditions, the stimulus consisted of separate ‘blue’ (RGB = [0 0 128]) and ‘green’ (RGB = [0 128 0]) discs. The discs in the two channels had the same spiral motion, but were separated horizontally by a constant offset as described in the text. Note that the blue disc matches background luminance in the blue channel and is therefore invisible through the blue filter but is darker than the background in the green channel and can therefore be seen through the green filter. The converse is true for the green disc.

During the experiment, mantises were presented with ten trials in each of the three disparity conditions, randomly interleaved, for a total of thirty presentations. There was a pause of 60 seconds between presentations of the stimuli. We ran the experiment at three viewing distances: 5, 7 and 10 cm. We tested six mantises at a viewing distance of 5 cm and four each at 7 cm and 10 cm. The size of the disc on the screen was increased with the physical viewing distance (Table 1, Fig. S1), so that in each case it subtended a visual angle of 22.6^o^ when at the center of the screen. This is the angle subtended by a physical disc of diameter 1 cm viewed at 2.5 cm. The disparity between the images visible in each eye was calculated to simulate a target at a distance of 2.5 cm from the mantis using a mantis interocular distance of 0.4 cm. Thus, the physical separation between the images ranged from 0.4 cm when the viewing distance was 5 cm, to 1.2 cm when the viewing distance was 10 cm. Since the disparities were always equal to or greater than the interocular distance of the mantis, the images in the uncrossed condition are divergent.

## Additional Information

**How to cite this article**: Nityananda, V. *et al.* Insect stereopsis demonstrated using a 3D insect cinema. *Sci. Rep.* **6**, 18718; doi: 10.1038/srep18718 (2016).

## Supplementary Material

Supplementary Information

Supplementary Video S1

Supplementary Video S2

Supplementary Video S3

Supplementary Video S4

## Figures and Tables

**Table 1:** Dimensions of the stimuli in the illusory depth experiment.

Viewingdistance	Diameter of disc stimulus		Stimulus disparity incrossed-disparity condition
	on screen	in visual angle	separation on screen
5 cm	2.0 cm(74 pixels)	22.6^o^	0.4 cm(15 pixels)
7 cm	2.8 cm(104 pixels)	22.6^o^	0.72 cm(27 pixels)
10 cm	4.0 cm(148 pixels)	22.6^o^	1.2 cm(45 pixels)
