# Supplementary material for: Insect stereopsis demonstrated using a 3D insect cinema
Source: Sci Rep. 2016 Jan 7;6:18718. doi: 10.1038/srep18718 (PMC4703989; doi:10.1038/srep18718)
Supplement: Supplementary Information [file srep18718-s1.doc]

# Insect stereopsis demonstrated using a 3D insect cinema: Supplementary Information

Vivek Nityananda^1*^, Ghaith Tarawneh^1^, Ronny Rosner^1^, Judith Nicolas^1, 2^, Stuart Crichton^1^, Jenny Read^1^

1. Institute of Neuroscience, Henry Wellcome Building for Neuroecology, Newcastle University, Framlington Place, Newcastle Upon Tyne, United Kingdom, NE2 4HH
2. M2 Comportement Animal et Humain, École doctorale de Rennes, Vie Agro Santé, University of Rennes 1, 35000, Rennes, France

Supplementary material: Circular polarization

Even in a monochromatic species, anaglyph 3D – separating the two eyes’ images spectrally – has the drawback that it is non-trivial to equalize the effective brightness of the two images. This would require correction for the emission spectra of the monitor primaries, the transmission spectra of the filters, and – most challengingly – the spectral sensitivity of the species. In humans, a common alternative is separating the two eyes’ images via circular polarization. The images then appear nearly equally bright without correction. We examined this technology in our mantis model, but found it failed to produce an illusion of 3D, apparently due to high levels of crosstalk. We document these experiments in this Supplementary Material. Methods are the same as in the main paper except as described below.

## Stimuli and display

Stimuli were displayed on an LG D2342 3D LCD monitor (<http://www.lg.com/us/monitors/lg-D2342P-PN-led-monitor>) with a resolution of 1920 x 1080 pixels, 54.58 cm wide x 34.19 cm high. This uses a film-type patterned-retarder in which alternate pixel rows are circularly polarized either clockwise or counter-clockwise. When the monitor is viewed through passive 3D glasses whose lenses are circularly polarizing filters of opposite handedness, each eye sees only the odd or only the even pixel rows. The image therefore appears in line-interleaved 3D. The 3D mode of the monitor was not used; the monitor was run in its 2D mode and the “line-interleaved stereo” mode of Psychophysics Toolbox was used to present stimuli in the two polarization channels (corresponding to odd and even pixel rows). This avoids artefacts caused by monitor properties intended to optimize human viewing of 3D movies.

## Preparation and fixing of 3D glasses

3D glasses were attached to the mantis as before, except that now these were cut out of Real 3D® glasses that are used for 3D film viewing at the cinema. Although not the LG glasses supplied by the manufacturer, these are the same kind of filters and, as our physical crosstalk measurements (Fig. S2) show, work equally well. These filters consist of a quarter wave plate combined with a linear polarizer, and are directional, i.e. they only work when the light from the monitor passes first through the quarter wave plate and then through the linear polarizer. Care was taken to ensure that the glasses were put on facing outwards, i.e., in the manner they would be worn when viewing a film at the cinema. Correct orientation could easily be verified by viewing the mantis through a pair of intact Real 3D glasses and closing one eye at a time. One lens of the mantis’ glasses should then appear dark, as the light transmitted by it is blocked by the experimenter’s own lens. Swapping which eye is closed should swap which lens of the mantis appears dark.

## Assessing interocular crosstalk

### Physical crosstalk measurements

A filter of one or the other polarity was fixed directly in front of a Konica Minolta CS-2000 spectrophotometer such that the outward facing surface when the 3D glasses were worn now faced the monitor, i.e., as if the spectrophotometer was ‘wearing’ the filter. Measurements were subsequently made in complete darkness. The monitor was white, i.e. at its maximum luminance, except for a gray square that covered the entire measurement area of the spectrophotometer. The luminance of the square was varied by increasing the digital driving level from 0 to maximum in 25 equal steps. At each step, we measured the luminance of the square as viewed through the filter. The experiment was run twice for each filter with the square first being presented in one buffer and then in the other, i.e. with one polarization orientation and then in the opposite. We then repeated the measurements with the filter of the opposite polarization. We repeated the measurements with the monitor first tilted forwards at an angle of 11° and then with the monitor flat but tilted horizontally at an angle of 15° to measure the crosstalk of the filters at oblique angles. These are the maximum angles at which the mantis would have viewed the stimuli once the stimuli had come to rest in front of the mantis at the center of the screen, with the horizontal angle slightly greater due to the horizontal disparity in the crossed and uncrossed disparity conditions. We measured crosstalk in all conditions by dividing the luminance transmitted through the filters when the stimulus was present in the channel opposite to the polarization of the filter by the luminance transmitted through the same filter when the channel and the filter were of the same polarization.

### Electroretinograms

Experiments were run on two different mantises in a manner similar to that for the spectral crosstalk measurements. The stimuli consisted of flashes presented in one or the other circular polarization channel of the 3D monitor, viewed through the matching or opposite filter.

### Behavioral measurements

In order to measure the crosstalk of the polarization filters behaviorally, we cut out two square (side = 2 cm) filters from Real 3D glasses. Mantises were placed at a viewing distance of 3 cm from the screen and two filters of the same polarization (both cut out from the same filter – left or right - of the 3D glasses) were held in a custom built holder directly in front of the mantis. Care was taken to ensure that both filters were facing the same way and were in the same orientation. The filters covered the entirety of the mantis’s view of the monitor. The background luminance of the monitor was kept constant at half the maximum digital driving level, i.e. 128 in all 3 primaries and in both polarization channels. We began the experiment after verifying the motivation of the mantis by presenting them with the stimulus at highest contrast subtending the same angle as during experiments and ascertaining that they struck at this stimulus. During the experiment, we presented a dark “swirling disc” stimulus in either one of the polarization channels of the monitor, or in both. The disc subtended 22.6^o^. As before, the contrast of the target was varied: the digital driving level of all 3 primaries in the given polarization channel was set to T=0, 26, 51, 77 or 102. The 5 contrast and 3 channel conditions of each stimulus were each presented 3 times, randomly interleaved, for a total of 45 trials per experimental run. There was a pause of 60 seconds between each presentation. This was to prevent any habituation effects and consequent loss of motivation of the mantis. We repeated the experiment with filters of the opposite polarity, testing five mantises with filters of one polarity and four with the opposite. All experiments were run in darkness, so that almost all the light reaching the mantis was from the monitor.

## Trying to present illusory 3D depth

### Test stimulus 1

The first stimulus consisted of a black (RGB = [0 0 0]) dot moving erratically on a white (RGB = [1 1 1]*maximum) background for ten seconds. The movement of the dot was restricted to a region directly in front of the mantis. The dot subtended an angle of 11.4° at the position of the mantis. This corresponds to the angle subtended by a target of 0.5 cm diameter when viewed at a distance of 2.5 cm. When the experiment was run with a viewing distance of 5, 10 and 20 cm this corresponded to a diameter on the screen of 1, 2 and 4 cm respectively. This target was presented in all three disparity conditions with the disparity simulating a depth of 2.5 cm in the crossed disparity condition and the equal but opposite disparity in the uncrossed disparity condition. This corresponded to a parallax on the screen of 0.4, 1.2 and 2.8 cm for viewing distances of 5, 10, and 20 cm respectively.

### Test stimulus 2

The second stimulus consisted of a black (RGB = [0 0 0]) dot on a grey (RGB = [0.5 0.5 0.5] *maximum) background. The dot spiraled in rapidly from the periphery of the screen on a previously specified path and ended directly in front of the mantis. The swirl lasted for a duration of five seconds after which the dot stayed in front of the mantis with a subtle jerky motion for two more seconds before vanishing. Apart from the display technology, this stimulus is identical to the stimulus used for the anaglyph experiments. The disparities used during display were as described for test stimulus 1 above but the angle subtended by the stimulus at all viewing distances was 22.6°. This corresponds to the angle subtended by a target of 1 cm diameter when viewed at a distance of 2.5 cm. The crossed disparity condition was thus simulating a target of 1 cm diameter at a depth of 2.5 cm from the mantis.

For both stimuli, we verified the motivation of the mantises before the experiments were run by presenting them with the stimulus subtending the same angle as during experiments but at a viewing distance of 2.5 cm. Once the mantis struck at this stimulus, we moved the mantis to the appropriate viewing distance and began the experiments, during which we presented the mantis each of the disparity conditions interspersed in random order. There was a pause of 60 seconds between each presentation during which the mantis only saw the background. A total of 30 presentations were made with each condition presented ten times. All experiments were run in darkness, so that most of the light reaching the mantis was from the monitor. Five mantises were tested with stimulus 1 and four mantises were tested with stimulus 2.

## Results

Fig. S2 shows the results of the crosstalk measurements. The crosstalk implied by luminance measurements is as expected for this type of stereoscopic display technology: 3-4% (Fig. S2a). The crosstalk implied by the electroretinograms is also low: < 10%. (Fig. S2b) though perhaps somewhat higher than the crosstalk observed with the colored filters (Fig. 2). The behavioral crosstalk, however (Fig. S2c), is disastrously high. There is virtually no evidence that the circular polarization is succeeding in separating images for the two eyes. Insects respond almost as often when the stimulus is presented in the “wrong” channel as in the correct one, and they respond much more strongly when it is presented in both channels. Thus, these results strongly imply that the mantises can see images equally well in both polarization channels, even when viewing the image through a filter intended to block one channel.

It is not surprising, then, that we also found 3D glasses created from these filters failed to produce a depth percept. Fig. S3 shows the results with polarizing 3D glasses. For test stimulus 1, we found that the mantis tracked but did not strike at the target. There was no significant independent main effect of disparity condition (Fig. S2c: Repeated measures ANOVA, F(2,8) = 4.3, Partial η^2^ = 0.518, P = 0.054), but there was a significant main effect of viewing distance (Repeated measures ANOVA, F(2,8) = 13.48, Partial η^2^ = 0.771, P = 0.003) and a significant interaction between viewing distance and condition (Repeated measures ANOVA, F(4, 16) = 0.075, Partial η^2^ = 0.577, P = 0.006). This is because mantises were much less likely to track the crossed-disparity stimulus when the monitor was 20 cm away from them. For test stimulus 2, the mantises did strike at the target but there was no difference in the mean proportion of strikes across the three disparity conditions (Fig. S2e: Repeated measures ANOVA, F(2,6) = 3.695, Partial η^2^ = 0.552, P = 0.09). Taken together, these results – especially the lack of any significant difference between the uncrossed and crossed conditions - fail to indicate convincingly that the mantises were using disparity cues to make their decisions on whether to track or strike at prey-like stimuli and therefore to make their depth judgments.

Stereoscopic stimuli displayed on this type of 3D monitor do have a vertical disparity. For humans, this is usually very small at the viewing distances used, and can be corrected by small reflex vertical vergence movements. For mantises, the viewing distance was much closer, making the vertical disparity much larger in angular terms, and mantises cannot make vergence movements to null the vertical disparity. Vertical disparity does impair mantis stereoscopic depth perception, just as in humans, but only when it exceeds around 12-15°^38^. The vertical disparity between odd and even pixel rows is 0.73° at a viewing distance of 2.5 cm. Thus, vertical disparity seems unlikely to account for the lack of depth perception. As noted, the likely explanation is the extremely high effective levels of interocular crosstalk.

It is unclear why the behavioral crosstalk is so much higher than indicated by our physical and physiological measurements (Fig S2). It is important to point out that the stimuli were very different in the three experiments. For the physical measurements, the stimuli were static squares covering the photometer’s measurement field. For the physiology, the stimuli were flashed squares at the center of the mantis’s field of vision. For the behavior, the stimuli were dark disks which spiraled in from the edge of the screen towards the center. One well-known drawback of this type of patterned-retarder polarization-based display is that crosstalk depends strongly on viewing angle, so we considered whether this could be a factor. The “swirling disc” stimuli in our experiments were designed to come to rest directly in front of the mantis, where crosstalk should be minimal, but the line of sight to the top or side of the disc is necessarily oblique: some 11^o^ or 15^o^ respectively (given the horizontal disparity). We measured the physical crosstalk at these oblique angles to see if this could account for our results, but the physical crosstalk always remained less than 10% (Fig. S4). Thus, this seems unlikely to account for our results if strikes were triggered purely by the final position of the disk. However, the whole reason for developing our “spiraling disc” stimulus was that strikes do not depend purely on the final position, but are more likely to occur if the insect sees a stimulus approach from the edges of the screen (even though the strike is released only when the stimulus is close to directly in front of the mantis). Clearly, viewing angles are much more oblique for stimuli at the edges of the screen. We did not measure crosstalk at oblique angles >15^o^, but we know that crosstalk with this type of patterned-retarder 3D display increases substantially with oblique viewing^21^.  Thus, in the “wrong channel” condition, the mantis likely saw an initially high-contrast disk moving in the periphery, becoming lower contrast as it spiralled in and nearly vanishing as it came to rest in front of the mantis. Apparently, this stimulus elicits as many strikes as a spiraling disc that remains high contrast throughout, as in the “correct channel” condition.

We also considered alternative explanations. The physical crosstalk measures we have reported for the circularly polarizing filters used the photometer’s luminance measurements. These reflect spectral radiance weighted for human, not mantis, photopic sensitivity. We considered whether there could have been substantially more crosstalk in regions of the spectrum that mantises are more sensitive to than humans, such as in the blue range (see Fig. S5a). However, when we used a spectrophotometer to measure crosstalk across the spectrum weighted by the mantis’s spectral sensitivity, we found no evidence of an increase in crosstalk (Fig. S5b). This is also borne out by additional experiments that measured crosstalk behaviorally when presenting the polarized stimuli only in the green primary of the LCD screen with the polarization filter taped to the screen covering the mantis’s entire view of the stimulus at the center of the screen. These experiments still showed high levels of crosstalk, indicating that the crosstalk was not restricted to the blue range of the spectrum (Fig. S5c).

It is natural to wonder whether mantis vision is somehow sensitive to polarization, but we have been unable to come up with a possible mechanism that could explain our results. The light which enters the mantis’ eyes after passing through the 3D filters is linearly polarized. Some insects are sensitive to linear polarization, although mainly in the dorsal rather than frontal regions of the eyes^39^. However, in the behavioral crosstalk experiments, a single large piece of filter was placed in front of the insect, covering the stimulus in both eyes. Light entering both eyes would therefore be linearly polarized in the same way. The polarization state of light entering the eyes should be the same regardless of what channel the stimulus was presented in; the contrast should simply be reduced for stimuli presented in the wrong channel. Thus, it is hard to see how any sensitivity to polarization state could explain our results. Our interpretation is that polarizing 3D glasses fail for stimuli which are not directly in front of the mantis because the oblique viewing angle causes unworkably high crosstalk.

# Supplementary figures

Figure S1. Binocular geometry showing how disparate stimuli on-screen imply a virtual target in front of the screen. The shaded regions show the extent of the dark target in each eye. The angle subtended by the target depends on its on-screen diameter (here labeled for the left eye) and on the physical viewing distance. The location of the virtual target depends on the stimulus disparity, which is the offset between the discs presented to left and right eyes. Where the discs overlap on the screen, as here, the overlap region appears black and the disparity corresponds to the size of the colored region.


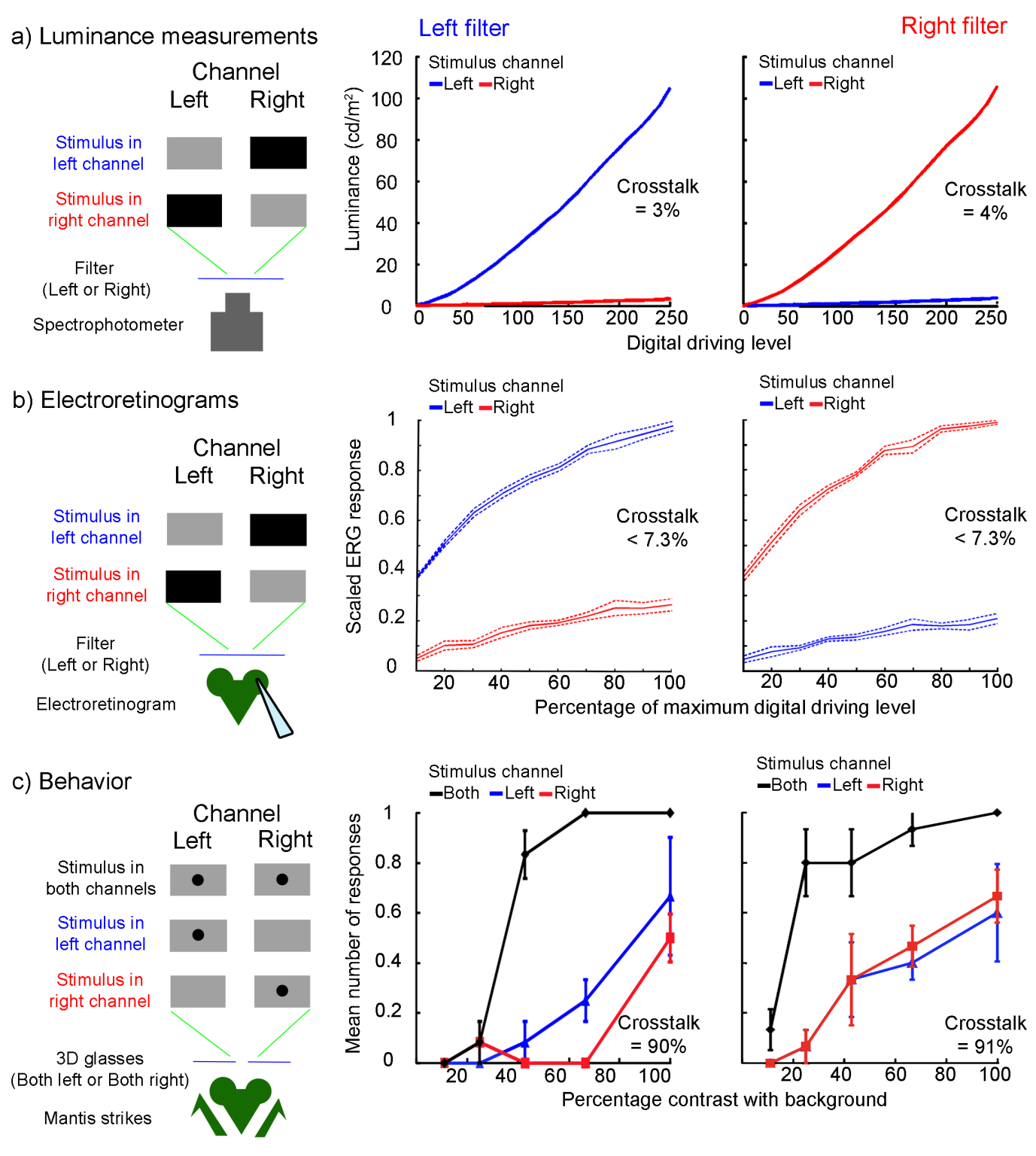


Figure S2: Polarization crosstalk measurements. Columns correspond to the different filter types – ‘left’ or ‘right’ polarity. Lines represent measurements when light was output in either the left (blue) or right (red) polarity buffer. a) Physical luminance and crosstalk measurements b) Electroretinograms in response to light flashes of different intensities viewed through one or the other filter when present in the channel with the same or opposite polarity or in both. The electroretinogram data represent the mean response and the standard error. c) Mean number of responses (strikes + tensions) to swirling stimuli present in the ‘left’ (blue line and triangles) or the ‘right’ (red line and squares) polarization channel or both (black line and diamonds).


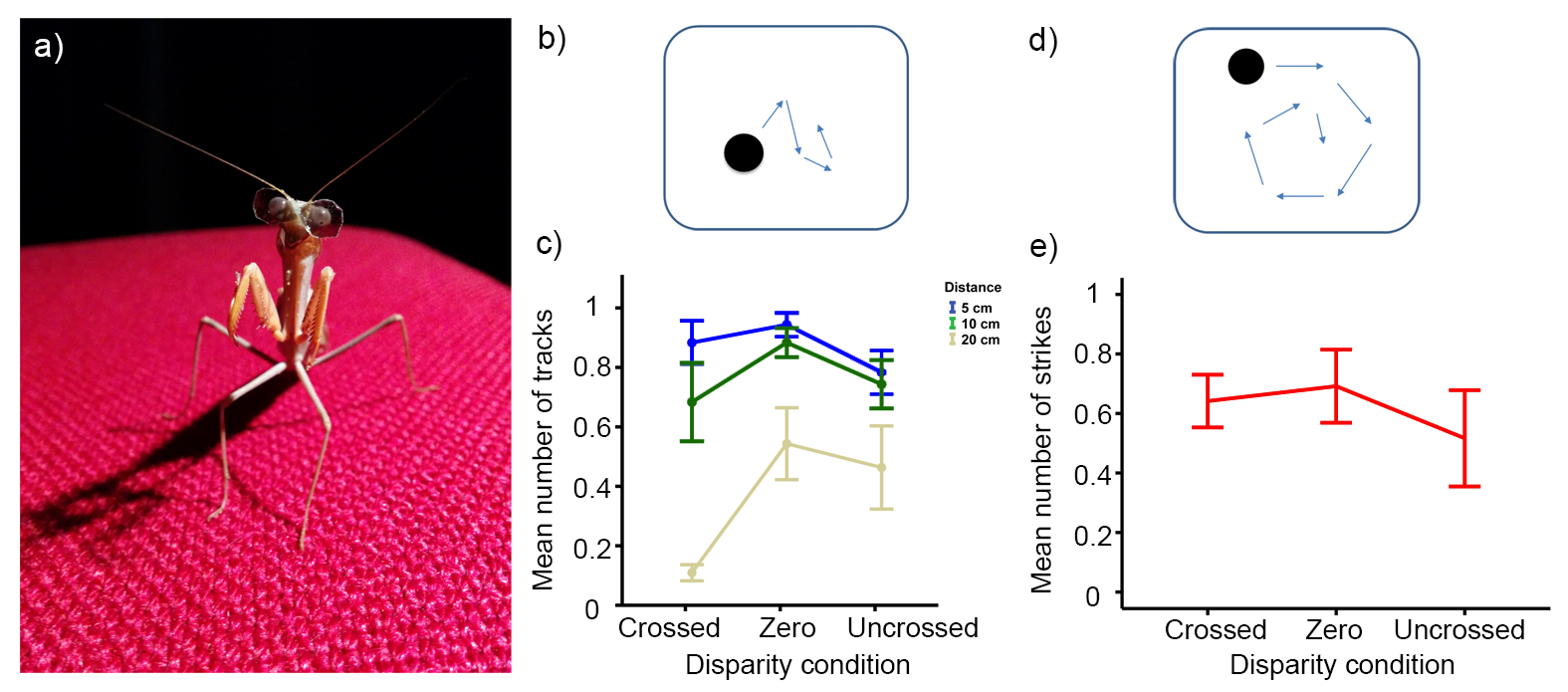


Figure S3: Polarization-based insect 3D. a) Mantis with polarized glasses. b) Stimulus used and c) responses observed for the first experimental test in the polarization based insect cinema across different disparity conditions and viewing distances (blue line = 5 cm; green line = 10 cm; beige line = 20 cm). Data represent mean number of tracks per trial and standard error. d) Stimulus used and c) responses observed for the second experimental test across different disparity conditions. Data represent mean number of strikes per trial and standard error.


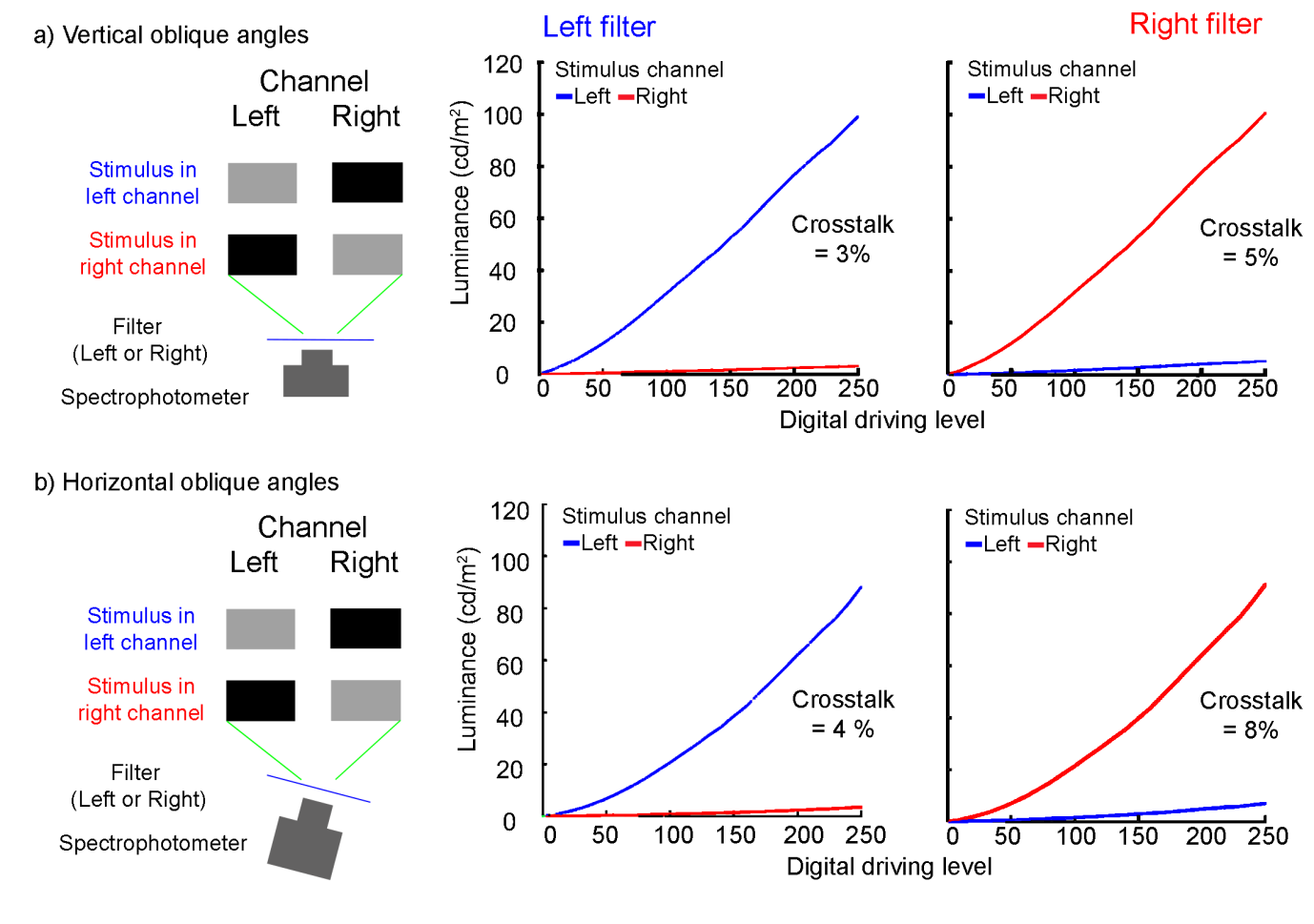


Figure S4: Physical luminance and crosstalk measured at a) a vertical oblique angle of 11° and b) a horizontal oblique angle of 15°. Columns correspond to the different filter types – ‘left’ or ‘right’ polarity. Lines represent measurements when light was output in either the left (blue) or right (red) polarity buffer.


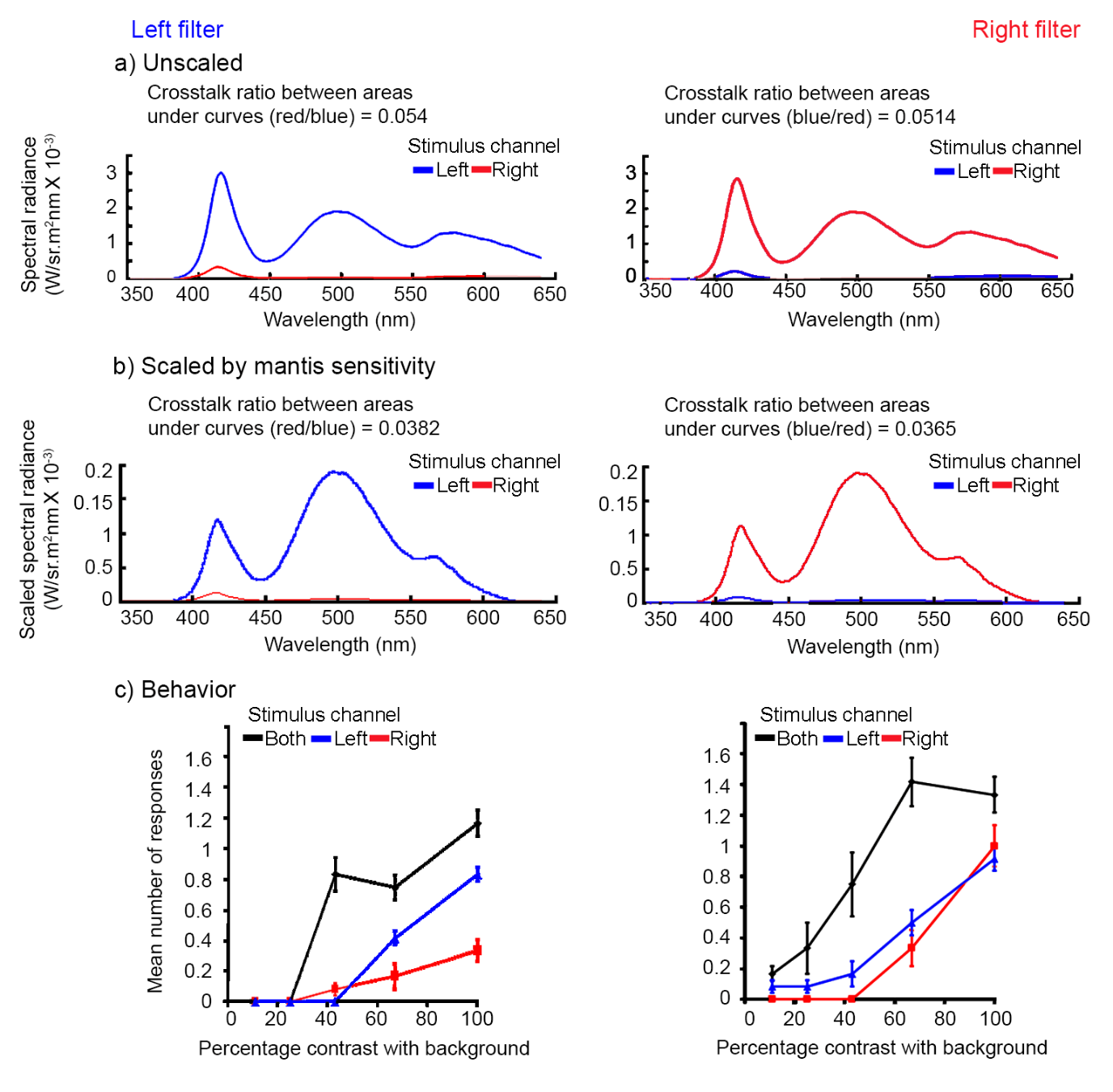


Figure S5: Spectral influence on polarization crosstalk. Columns correspond to the different filter types – ‘left’ or ‘right’ polarity. a) Measured radiance across the spectrum of right (red curve) and left (blue curve) polarized light through the right and left filters and b) after multiplying the value at which wavelength by the sensitivity function of the mantis (see Fig. 1). Crosstalk measurements given were made by dividing the area under the curve for the signal that didn’t match the filter by that of the filter that did. c) Mean number of responses (strikes + tensions) to swirling stimuli output using only the green primary in the ‘left’ (blue line and triangles) or the ‘right’ (red line and squares) polarization channel or both (black line and diamonds).

**Supplementary Videos**

Videos S1-S3: Exemplar videos of a mantis responding to stimuli presented in the crossed (S1), uncrossed (S2) and zero (S3) disparity conditions. Note that these are not the actual videos used in analysis which were recorded so that the stimulus was not visible.

Videos S4-S6: Exemplar high-speed videos of a mantis responding to stimuli presented in the crossed (S4), uncrossed (S5) and zero (S6) disparity conditions. Note that these are not the actual videos used in analysis which were recorded so that the stimulus was not visible.
